# Arthur James Macdonald Bentley

**Published:** 1911-06

**Authors:** 


### ARTHUR JAMES MACDONALD BENTLEY, M.D., C.M.

We regret to learn of the sudden death from hemorrhage at Llandrindod Wells, on April 19th, at the age of 61, of Dr. Arthur Bentley, who gave a course of lectures on Tropical Diseases in the University of Bristol, 1910. He was formerly Colonial Surgeon at the Straits Settlements, and accordingly spent all his early medical life at Singapore, where he learnt much of tropical maladies. Recently his time was divided between Egypt, where he was well known as Physician to the Mina House Hotel at Cairo, and for the last few years at Llandrindod Wells in the summer months. He was appointed as lecturer in Tropical Diseases in the Universities of Manchester and Bristol, and was the author of several treatises on Beri-Beri, Elephantiasis and matters relating to health in Egypt and Llandrindod Wells.

Those who had the privilege of his friendship must feel that they have lost a genial companion, whose experience of life had been large, and from whom there was always much to be learnt. He wrote also the following contributions: "Elephantiasis, with Special Relation to Pathology of Lewis" (*Lancet*, 1878, i, 784), "On Warburg's Tincture" (*Gen. Remed. Tijds.-Ned. India*, 1874), and "Helouan as a Health Resort" (*The Health Resort*, 1905).

